# Cerebrospinal fluid protein levels are elevated 100 times in a Leptomeningeal metastasis patient: a case report and literature review

**DOI:** 10.3389/fnins.2023.1174309

**Published:** 2023-05-17

**Authors:** Shengnan Wang, Wenzhuo Yang, Mingqin Zhu, Xiaochuang Wang, Lin Pan, Tao Jin, Youqi Chen, Jianxin Xi, Laiyu Yang, Run Cui

**Affiliations:** ^1^Department of Neurosurgery, Guangdong Second Provincial General Hospital, Guangzhou, China; ^2^Department of Neurology, The First Hospital of Jilin University, Changchun, China; ^3^Department of Neurosurgery, Cancer Hospital of Sun Yat-sen University, Guangzhou, China; ^4^Clinical College, Jilin University, Changchun, China

**Keywords:** leptomeningeal metastasis, tuberculous meningitis, CSF protein, cytology, CNS tumor

## Abstract

Leptomeningeal metastasis (LM) has a high degree of malignancy and high mortality. We describe a patient admitted to hospital with acute lower extremity weakness, dysuria, and high intracranial pressure. Enhanced magnetic resonance imaging (MRI) showed extensive enhancement of the leptomeningeal and spinal meninges with multiple nodular changes and extensive fusion. His cerebrospinal fluid (CSF) was yellow and cloudy, the Pandy test was strongly positive (++++), the protein was 46 g/L (normal range 0.15–0.45 g/L), which attracted our attention. Initially, miliary TB with associated tuberculous meningitis (TBM) was diagnosed, and neurosarcoidosis cannot be ruled out. After poor therapeutic effect of standard antituberculosis (anti-TB) therapy, further inspection found that malignant cells were detected by cerebrospinal fluid (CSF) cytology. PET/CT suggested the diagnosis of LM. The purpose of this paper is to describe the characteristics of atypical diffuse LM. In conclusion, when patient with unexplained high levels of CSF protein, it is necessary to be alert to the diagnosis of LM. Multiple examinations of fresh CSF are helpful to increase the positive detection rate of tumor cells. Early diagnosis and active treatment are conducive to improving survival rate.

## 1. Introduction

Leptomeningeal metastasis (LM) or leptomeningeal carcinomatosis, is characterized by the spread of tumor cells to the leptomeninges and the subarachnoid space (Mack et al., 2016). When LM is diagnosed, it is usually late in the disease process, and it is usually associated with a high level of systemic tumor burden (Wasserstrom et al., 1982; Balm and Hammack, 1996). Patients can present with a wide range of symptoms related to the involvement of various sites in the craniospinal axis. It is not always easy to diagnose, and clinicians need to always have a high level of suspicion.

Protein levels in cerebrospinal fluid (CSF) are one of the most sensitive indicators of pathology in the central nervous system. CSF protein levels are elevated in infections, intracranial hemorrhages, multiple sclerosis, Guillain Barré syndrome, malignancies, some endocrine abnormalities, certain medication use, and a variety of inflammatory conditions (Talati et al., 2008).

Non-tuberculous mycobacteria (NTM) infections of the CNS are extremely rare. Neurosurgery, trauma, intracranial implants, otomastoiditis, and disseminated infection are important modes of acquisition (Maniu et al., 2001). Infections due to Mycobacterium abscessus complex are rare among NTM CNS diseases, and only four case reports have been published in English-language literature (Liebeskind et al., 2001; Maniu et al., 2001; Seehusen et al., 2003; Lee et al., 2012).

Here, we present a case of a patient without a history of systemic malignancy, who was diagnosed with LM after a delay. The patient's CSF protein was extremely high (46.17 g/L, normal range 0.15–0.45 g/L) and glucose was extremely low (0.28 mmol/L, normal range 2.3–4.1 mmol/L), which attracted our attention. Since no cancer cells were found in the CSF, and the MRI features were similar to those of tuberculous meningitis, the patient was diagnosed as tuberculous meningitis at an early stage of onset. The patient did not improve after standard antituberculosis (anti-TB) treatment. After repeated CSF examinations, heteromorphic cells were discovered, and the patient was eventually diagnosed with LM.

## 2. Case report

**Figure 1:**
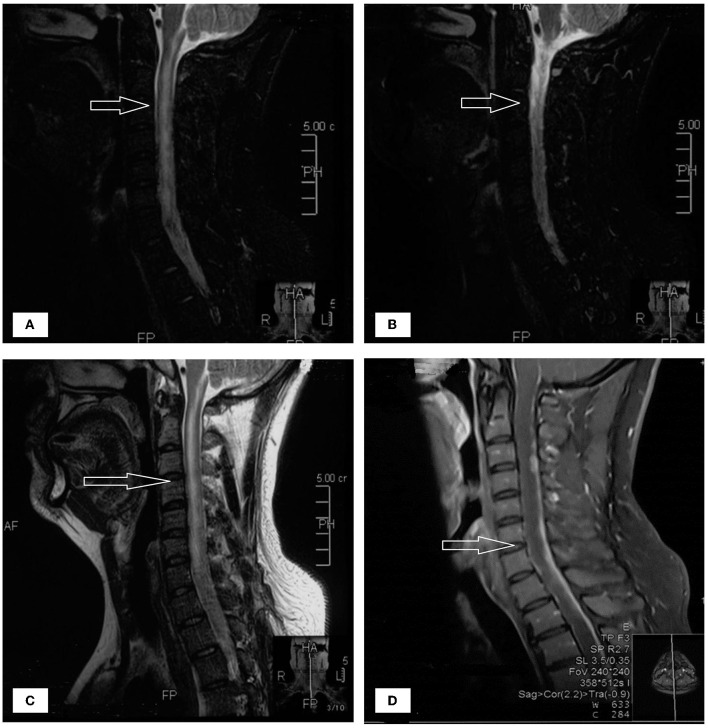
**(A–D)** The patient's spinal MRI showed multiple abnormal signals in the central canal of spinal cord, as shown by the arrow.

A 33-year-old male who had been experiencing neck pain for a month and dysuria for a week was admitted to a local hospital for examination. Cervical MRI plain scan and enhanced scan done at the local hospital revealed multiple abnormal signals in the central canal of spinal cord on November 28, 2021 (Figure 1). Primary lumbar puncture (LP) was performed, and his CSF suggested high protein (2.95 g/L, normal range 0.15–0.45 g/L), high white blood cells (WBC) count (243^*^10^6^/L, normal range 0–8^*^10^6^/L), low glucose level (0.88 mmol/L, normal range 2.3–4.1mmol/L) and low chloride level (113 mmol/L, normal range 119–129 mmol/L). Color Doppler sonography of urinary system suggested right renal cyst, turbid urine in the bladder, prostate calcifications and prostate cyst. Ceftazidime was given anti-inflammatory treatment during local hospitalization, but he did not show improvement and had trouble defecating after 2 days. On the third day the patient was transferred to our hospital for further treatment.

The patient had dizziness, headache, nausea and vomiting occasionally, however, no physical activity disorder and no fever was present during the early stage. The patient was previously healthy and had occasional neck pain a year ago, which resolved spontaneously. Neurological exam showed right lower limb muscle strength grade 4, Kernig sign (+), neck rigidity (+), and a bilateral Babinski sign, all other neurological examination were otherwise normal. The brain MRI were conducted on the second day following admission, shows abnormal signal and nodular enhancement of medulla oblongata and cervical medulla. Considering the patient's long spinal cord lesion, mannitol Q6H dehydration treatment was given at once. Upon admission, based on the clinical symptoms, the possibility of myelitis was considered. Levels of AQP4, MOG, MBP and GFAP antibodies in serum and CSF were measured and negative.

**Figure 2:**
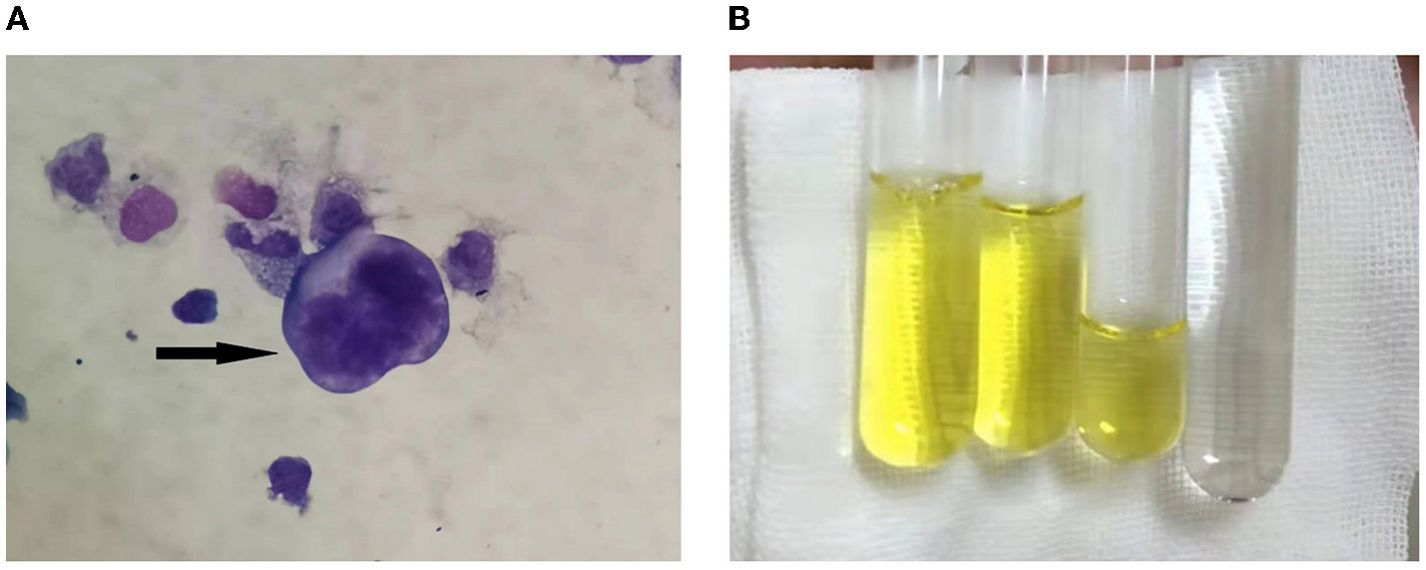
**(A)** Cerebrospinal fluid cytology showed atypical tumor cells, as shown by the arrow. **(B)** The CSF aspired was yellowish and viscous compared with the clear water.

We performed LP to collect CSF on the third day following admission and yielded a surprising result. CSF routine test showed high intracranial pressure (240 mmH_2_O, normal range: 80–180 mmH_2_O). CSF was yellow and cloudy (Figure 2B), the Pandy test was strongly positive (++++), the protein was 46 g/L (normal range 0.15–0.45 g/L), which was an unbelievable result. His CSF glucose was also extremely low (0.28 mmol/L, normal range 2.3–4.1 mmol/L), which was a significant deterioration compared to the last test performed a week ago. Cytology was not possible due to rapid coagulation of the patient's cerebrospinal fluid.

The patient's condition deteriorated rapidly. Combined with the imaging findings and extremely elevated cerebrospinal fluid protein levels, tuberculous meningitis cannot be ruled out. Standard antituberculosis (anti-TB) therapy was given, including isoniazid 0.6 g 1/day intravenously, rifampicin 0.6 g 1/day intravenously, pyrazinamide 1.5 g 1/day orally and ethambutol 0.75 g 1/day orally. At the same time, liver protection treatment was carried out. Two days later, he had vision and restlessness at night, which was relieved by risperidone. The next morning, the patient developed severe headache and projectile vomiting. Neurological exam showed double lower limb muscle strength down to grade 3. We performed the third LP to collect CSF. His intracranial pressure was over 400 mmH_2_O. CSF routine test showed the protein was 34.12 g/L, the glucose was 0.15 mmol/L, the WBC count was 202^*^10^6^/L and the Chloride level was 102.4 mmol/L. Metagenomic next-generation sequencing (NGS) of viral and bacterial genomes from the CSF was performed, which was positive exclusively for the normal skin flora DNA and was not significant. Xpert MTB/RIF prior, rpoB sequencing and Brucella serology were also performed, but no significant results were detected. A day later, the patient was transferred to the infectious disease hospital for further treatment.

CSF cytology specimens were performed twice and were finally positive for malignant cells (Figure 2A). We performed whole genome sequencing for MTB&NTM and full-length resistance genes sequencing by the third generation nanopore sequencing technology. Subsequently, mycobacterium abscessus have been sequenced, the number of which was 7,940. He subsequently underwent the whole-body 18F-FDG positron emission tomography/computed tomography (PET/CT) scanning. It revealed multiple hypermetabolic foci in leptomeningeal and the whole spinal cord, indicating primary meningeal malignancy with spinal cord spread. The patient was eventually diagnosed with LM and admitted to the Radiotherapy Department of our hospital on December 24, 2021. Although we actively contacted neurosurgery department of our hospital to prepare the spinal cord biopsy, the patient had a fever and died after 2 days.

## 3. Discussion

We present a 33-year male patient, who manifested as acute episode of dysuria and lower limb weakness with marked inhomogeneous enhanced nodules in the meninges. Tuberculous meningitis was initially considered, anti-TB and dehydration therapy were given. However, the symptoms of the patient progressively deteriorated. Afterwards, malignant cells were found in his CSF, and mycobacterium abscessus 7,940 infection was detected, LM and CNS Mycobacterium abscessus infection was diagnosed. Unfortunately, the patient died without further diagnosis.

The patient presented with acute episode of dysuria and lower limb weakness with marked inhomogeneous enhanced nodules in the meninges. CSF protein levels are elevated up to over 100 times. Neuromyelitis optica spectrum disorders (NMOSD) scans may occasionally show a cloud-like pattern of gadolinium enhancement with an inhomogeneous appearance and poorly defined margins; also, enhancements of the peripendyma and leptomeninges are common (Pekcevik et al., 2016). A variety of infectious diseases can also cause cerebral enhancement, including Lyme disease, Candida albicans, Cryptococcus, neurotuberculosis, and histoplasmosis (Bot et al., 2020). Combined with the results of several routine examinations of CSF (Table 1), the patient was critically ill at that time, with a rapid progression of his condition. Thus, tuberculous meningitis was initially considered and the patient was treated with anti-TB and dehydration therapy while awaiting the return of NGS for viral and bacterial genomes results, however, the patient's symptom deteriorated with the above treatment and NGS results came back negative. Although NGS provides a substantial improvement in accurate diagnosis of TBM, it still had a negative predictive value of 90.1% for definite TBM, which does not represent a perfect rule-out test. Therefore, the diagnosis of tuberculous meningitis cannot be excluded, and we continued with LP examination to collect CSF for further diagnose.

**Table 1:** Cytological examination of CSF.

	**Protein (normal range 0.15–0.45 g/L)**	WBC count (normal range 0–8^*^10^6^/L)	**Glucose level (normal range 2.3–4.1 mmol/L)**	**Chloride level (normal range 119–129 mmol/L)**
December 1, 2021	2.95 ↑	243 ↑	0.88 ↓	113 ↓
December 7, 2021	46.17 ↑	106 ↑	0.28 ↓	105.5 ↓
December 9, 2021	34.12 ↑	202 ↑	0.15 ↓	102.4 ↓

↑, increase; ↓, decrease.

Symptoms of LM include cranial nerve palsies, radicular symptoms, and signs of increased intracranial pressure, such as headaches, nausea, vomiting, and cognitive dysfunction (Mack et al., 2016). Due to the rapid progression of the patient's disease, the possibility of tumor was considered. Meningeal metastases commonly present as arachnoidal, subependymal, or dural enhancement; superficial cerebral lesions; and communicating hydrocephalus (Bot et al., 2020). Therefore, LM may be the primary lesion in patients with malignant tumor, although the patient has no history of tumor. In addition to clinical manifestations of neurologic involvement and tumor history, LM diagnosis relies more on CSF cytology and imaging (especially MRI enhanced scanning). At present, finding tumor cells in CSF is the gold standard for LM diagnosis, but there is a certain false negative rate. Therefore, withdrawing a sufficient amount of CSF or repeating the procedure multiple times were recommended in order to avoid false-negative results (Glantz et al., 2015). Afterwards, malignant cells were found in his CSF, connecting with his PET/CT results, the diagnosis of LM was excluded.

Non-tuberculous mycobacteria (NTM) refer generally to mycobacteria other than Mycobacterium tuberculosis complex (MTB) and Mycobacterium leprae. Moreover, MTB and NTM infections often cause indistinguishable clinical symptoms, but their treatment can be vastly different (Peng et al., 2021). Clinically, the low prevalence of NTM infections puts them rarely in the differential diagnosis of CNS infections, which occurs more frequently in patients with immunosuppression. Mycobacterium abscessus is an acid-fast NTM, which can cause the most drug-resistant NTM infections (Kasperbauer and Groote, 2015), and there is an urgent need for new drug development to improve the treatment outcomes for NTM diseases (Koh, 2011; Choules et al., 2019). Antimicrobial susceptibility testing should be performed whenever possible to guide therapy selection. Therefore, additional attention should be paid to the screening and identification of patients with unexplained CNS infection in clinical practice.

Under normal circumstances, CSF protein levels are much lower than those in the blood, and in healthy people CSF protein levels are about 0.5% of plasma protein concentrations (normal range 35–55 g/L). The patient's CSF protein concentration reached an astonishing 46 g/L, which has never been reported in the literature before. We analyzed that the increased CSF protein content in patients with meningeal carcinomatosis is due to the massive proliferation of cancer cells infiltrating meningeal, which further destroys the blood-brain barrier, elevates vascular permeability, and leads to a large amount of exudation of WBC and proteins. The blood-CSF-barrier dysfunction in leptomeningeal metastasis is most likely caused by reduced CSF absorption due to obstruction by malignant cells (Chamberlain, 2008; Djukic et al., 2017). At the same time, the patient was complicated with NTM infection, which further aggravated the damage and increased permeability of BBB. Although the patient had a significant increase in CSF protein concentration, it did not lead to a definitive diagnosis of meningeal carcinomatosis, and CSF cytology remained irreplaceable. Unfortunately, the patient died too early, and we didn't have time to perfect the biopsy to determine the nature of the tumor, which was a limitation of our study.

LM is an advanced malignant tumor with poor prognosis. Untreated patients cannot relieve symptoms and the course of disease is irreversible. At present, the treatment effect of LM is not ideal, there is no clear treatment method, and there are still no standard treatment guidelines. The main purpose of the treatment of LM is to reduce and improve the patient's clinical neurological symptoms and signs, prolong the survival period, and improve the quality of life. Current treatment methods include surgery, radiotherapy, systemic chemotherapy, intrathecal chemotherapy, targeted therapy, immunotherapy and support therapy.

In conclusion, LM has a variety of manifestations, and the possibility of LM should be considered in patients with increased intracranial pressure, cranial nerve palsy, or spinal nerve root involvement accompanied by abnormal white matter signals in the spinal cord. Further enhanced cerebral spinal MR scan and CSF cytology are required to confirm the diagnosis. For patients with diagnosis difficulties, enhanced spinal membrane or nerve root biopsy is feasible when necessary to avoid delayed diagnosis and treatment and affect the prognosis.

## Data availability statement

The original contributions presented in the study are included in the article/supplementary material, further inquiries can be directed to the corresponding author.

## Ethics statement

Written informed consent was obtained from the individual(s) for the publication of any potentially identifiable images or data included in this article.

## Author contributions

SW drafted the article. LP, WY, and XW contributed to editing and revision. TJ contributed to patient follow-up. MZ and RC has substantively edited the manuscript. JX, YC, and LY collected clinical data for us. All author has made substantial contributions to the manuscript. All authors have read and agreed to the final version of this manuscript.
